# *In-vitro* Callus Propagation and Secondary Metabolite Quantification in *Sericostoma pauciflorum*

**Published:** 2012

**Authors:** Satish C. Jain, Boskey Pancholi, Renuka Jain

**Affiliations:** a *Medicinal Plants and Biotechnology Laboratory, Department of Botany, University of Rajasthan, Jaipur-302004, India.*; b *Department of Chemistry, University of Rajasthan, Jaipur-302004, India. *

**Keywords:** *Sericostoma pauciflorum*, Antimicrobial, Antioxidant, Phytochemical analysis

## Abstract

*Sericostoma pauciflorum *Stocks ex Wight (Family Boraginaceae) used against cancer, diabetes and known to be health promoter. Callus cultures have been established from the stem explants on Murashige and Skoog (MS) medium supplemented with 1.5 mg/L concentration of different growth hormone viz. kinetin (Kn), indole 3-acetic acid (IAA) and indole 3-butyric acid (IBA). At 6 weeks of age, these calli were harvested, dried and extracted successively in pet, ether, methanol and water. Extracts were dried, weighed (%) and analyzed for their bioefficacies. Antimicrobial activities were determined using agar well diffusion and antioxidant potentials by DPPH and FRAP methods. Among all the test extracts, the extract of stem callus raised on IBA found to be more effective whereas it’s pet. ether extract showed appreciable activity against both the test bacteria and fungi (*S. aureus- *IZ 14.00 ± 0.57 mm and *T. rubrum- *16.33 ± 0.32 mm), followed by methanol extract (*S. aureus- *IZ 13.00 ± 0.57 mm, *A. niger *and *P. chrysogenum*- IZ 16.66 mm in both). In antioxidant potentials, all aqueous extracts were more active where IBA and Kn extracts demonstrated 0.06 mg/mL IC50 value (% inhibition 93.30 and 92.70 respectively at 0.8 mg/mL concentration) with 366 ± 6.69 and 343 ± 3.34 ascorbic acid equivalent antioxidant potentials at 1 mg/mL concentration. Furthermore, the chemical profile of test extracts was carried out. The bioactive secondary metabolites, *β*-sitosterol and caffeic acid was isolated from culture tissue of 6 weeks-old callus, and their identification and confirmation was carried out by color reaction, TLC behavior and IR spectrum techniques.

## Introduction

*Sericostoma pauciflorum *Stocks ex. Wight, commonly known as “karvas”, is an important xerophytic medicinal herb. Genus *Sericostoma *have eight species distributed throughout the Tropical, North East of Africa and North West of India. It is a short straggling under-shrub, growing widely throughout sea coast of Saurashtra and Maharashtra and used in making an important drug in Ayurveda named “Krishnavalli”, which is used against cancer, diabetes, dehydration, acidity and known to be health promoter. The whole plant is extensively used in indigenous system of medicine (as described in “Nighantu Ratnakar”). The plant contains medicinally important secondary metabolites viz., *β*-sitosterol, leupeol, *α*-amyrin, *β*-amyrin, pauciflorinyl acetate, pauciflorol acetate and sericostinyl acetate that are known for anti-inflammatory, anticancerous, antibacterial and antioxidant activities (1-4).

Plant growth regulators are one of the most important factors affecting cell growth, differentiation and metabolite formation (5). The appropriate concentration of the medium is one of the critical determinants in controlling callus growth and metabolite production. To produce cell dry mass as well as secondary metabolites from medicinal plants, it is important to establish the optimal culture conditions (chemical and physical environments) for the plant species used. The individual levels of auxin and cytokinin in the media used influenced the growth and regulation of cell metabolism. In addition, oxidative stress also plays important role in the production of secondary metabolites in plants. Phenolic compounds are considered to be secondary metabolites that are synthesized in plants through the phenylpropanoid pathway and function as a defense mechanism that reacts to various biotic and abiotic stress conditions (6). The exposure of plants to unfavorable conditions leads to the generation of reactive oxygen species (ROS) (7).

No reports are available on the growth, secondary metabolites production, antioxidant and antimicrobial activities of cell cultures. In this relation, the aim of this work was to compare the effects of different growth hormone therein on the production of secondary metabolites along with antioxidant and antimicrobial activities. This could elucidate the dependence of accumulation of these compounds of secondary metabolism on hormonal composition of the nutrient medium.

## Experimental

All the chemicals and solvents used in this study were of analytical grade and obtained from HiMedia Chemicals Mumbai, India.

*Plant material*

The whole plants of *S. pauciflorum *were collected from the fields locally during July-October, 2008. The botanical identity was confirmed by Herbarium, Department of Botany, University of Rajasthan, Jaipur. (Voucher specimen NO. 110). The plant has been deposited at the Herbarium and Laboratory for further reference.

*Establishment of cell cultures*

*S. pauciflorum *was collected from the wild regions of Jaipur. Stem explants were excised from the young plant, washed in running tap water for 30 min, dissected into small pieces and then washed with 2% commercial grade detergent for 5 min. Surface disinfection was done by mercuric chloride (0.1% for 4 min) and thoroughly washed with sterile distilled water, 5 min for each wash. Sterilized stem cuttings were cultured on MS (8) medium consisting of basal salts and vitamins with 3% w/v sucrose and 0.8% agar supplemented with different growth regulators. Indole-3-acetic acid (IAA), Indole-3-butyric acid (IBA) and Kinetin (kn) at various concentrations (0.5, 1, 1.5 and 2 mg/L) were used. The pH of the medium was adjusted to 5.8 and autoclaved at 15 psi for 15 min. Cultures were maintained at 26 ± 2ºC under 16 h photoperiod illuminated by fluorescent light (2000-3000 lux) and 55 ± 5% relative humidity. Regenerated callus were sub-cultured after 6 weeks on the respective medium. Their growth indices and moisture contents were calculated. Callus of 6 weeks age was harvested and used for further study.

*Extract preparation*

Callus on different growth hormones was harvested (20 mg dry weight in each case) as the whole plant (including root, shoot and leaf), extracted successively in petroleum ether (pet. ether), methanol and water (60 ºC, 24 h). These extracts were filtered through Whatman filter NO. 1, dried, weighed and stored at 4ºC for further experiment.

*Phytochemical analysis*

Total phenolics were measured by following the method using Folin-Ciocalteu reagent (9). A stock solution of the standard phenol (gallic acid) was prepared in ethanol, out of which 0.1 to 0.9 mL was taken into separate test tube and raised to 1 mL of ethanol. To each tube, 2.5 mL of deionized water and 0.1 mL (2N) Folin-Ciocalteu reagent was added and allowed to stand for 6 min. Later, 0.5 mL of 20% sodium carbonate solution was added, incubated for 30 min and the absorbance was taken at 750 nm using UV- Vis spectrophotometer. Total phenolics were expressed as mg gallic acid equivalents (GAE/g dry weight).

Total flavonoids content was measured by the aluminum chloride colorimetric assay (10). A stock solution of standard quercetin was prepared in ethanol, out of which 0.1 to 0.9 mL was taken, raised to 1 mL with ethanol and added to 10 mL volumetric flask containing 4 mL of distilled water. To this, were added 0.3 mL 5% NaNO2, 0.3 mL 10% AlCl3 (after 5 min) and 2 mL of 1 M NaOH (after 1 min) in sequence. Total volume was made up to 10 mL with distilled water. The solution was mixed well and the absorbance was measured against the prepared blank reagent at 510 nm.

The extracts were applied on Thin Layer Chromatography (TLC; silica gel G coated plates) along with the standard reference compounds in air-tight chamber containing benzene : heptane : alcohol (100 : 100 : 1) for triterpenoids (pet. ether extract) and *n*-butanol : acetic acid : water (4 : 1 : 5; upper phase) for phenolics (methanolic extract). Identification of compounds was carried out by exposing the plates with I2 vapors, NH3 vapors and UV light chambers. Spraying of terpenoids was done with 20% H2SO4 and 10% SbCl3 separately, whereas spraying of phenolic compounds was through 1% methanolic AlCl3 and Folin-Ciocalteu reagent. Rf values of standard and samples were also calculated. The spots coincided to reference markers were scrapped from unsprayed plates, eluted with methanol, filtered, evoparated to dryness, reconstituted and also crystallized in methanol. The melting point of the isolated compounds was determined in capillary tubes (Toshniwal Melting point apparatus) and subjected to ir spectrum (Perkin Elmer 337, Grating Infrared spectrophotometer).

*Quantification of the compounds*

Quantification was carried out using PTLC. The levels of terpenoids were estimated colorimetrically by Das and Benergee (11) method. Optical density (OD) was measured at 540 nm against the blank. Standard curves of the identified compounds were prepared using 0.01-0.1 mg/mL concentration. Total levels of caffeic acid were determined using Folin-Ciocalteu colorimetric reagent (9). OD was taken at 760 nm using UV-visible spectrophotometer. Standard curve of caffeic acid was prepared using 0.01 to 0.01 mg/mL concentration using similar procedure.

*Antibacterial efficacy*

For antibacterial screening, pure cultures of test bacteria were used. Antimicrobial assay was performed by agar well diffusion method (12). Inoculum was prepared by suspending bacteria in Nutrient broth medium and fungus in Sabouraud dextrose broth medium overnight at 37ºC (106-107 CFU/mL concentration). Bacterial and fungal suspensions were inoculated in Nutrient agar and Sabouraud dextrose agar plates, respectively. Plates were then incubated at 37 °C for bacteria and 25ºC in case of fungi for appropriate time periods under aerobic conditions. The diameter of the inhibition zone around each well was measured and recorded by Inhibition Zone Recorder (HiMedia, India). Gentamycin (10 mcg/disc) and Ketonocozole (10 mcg/disc) were used as positive controls for bacteria and fungi, respectively.

*1,1-Diphenyl-2-picrylhydrazyl (DPPH) radical scavenging activity*

The effect on DPPH radical was determined using the method by Fogliano *et al. *(13). Different concentrations of extract (0.8, 0.6, 0.4, 0.2, 0.1 mg/mL) were prepared in methanol and mixed with 2.5 mL of DPPH (2 mg/10 mL methanol). After 30 min of incubation time, OD was measured at 517 nm. Quercetin was used as standard. OD was measured at 517 nm using UV-Vis spectrophotometer. Negative control (methanol) and positive control (quercetin) were also used. Capability to scavenge the DPPH radical was calculated using the following equation:

DPPH scavenging effect (%) = [(A0 - A1 / A0) × 100]

Here, A0 was the absorbance of the control reaction and A1 was the absorbance in presence of the sample of given extract.

*Ferric ion reducing antioxidant potentials (FRAP)*

Total reducing power of extracts was determined according to FRAP (Ferric ion reducing antioxidant potentials) method (14). Specific concentration of standard (ascorbic acid) and extract (62.5-1000 μg/mL) was prepared in 1 mL ethanol followed by the addition of 2.5 mL phosphate buffer (0.2 M, pH = 6.6) and potassium ferricyanide (1%). After 20 min of incubation (50ºC), 10% of trichloroacetic acid (2.5 mL) was added and the solution was centrifuged (1000×g) for 10 min. A volume of 2.5 mL of upper layer was taken and mixed with equal amount of distilled water followed by the addition of ferric chloride (0.5 mL; 1%), incubated for 30 min and OD was measured at 700 nm. A standard calibration curve of ascorbic acid (10-500 mg/mL) was prepared and the antioxidant activity was expressed in mg ascorbic acid equivalents (mg AAE/g) of the extract.

*Statistical analysis*

All the experiments were performed in triplicate, statistically analyzed and expressed as mean ± standard error (SE).

## Results and Discussion

During the present set of experiment, different growth regulators (IAA, IBA and Kn) on MS medium were tried for callus induction from stem nodal explants. Out of three hormone used, IAA and IBA regenerated callus were brownish and embryogenic in nature whereas Kn regenerated callus was greenish and non-embryogenic. Among all tested concentrations, MS medium supplemented with 1.5 mg/L growth hormone, produced compact, fast growing callus and was used for the further activity.

On total phenolics and flavonoids estimation, MS medium supplemented with Kn demonstrated higher levels of total phenolic contents, *i.e*. 107.33 ± 1.76 (mg GAE/g dw) followed by IBA supplemented callus (100 ± 5.78 mg GAE/g dw). On total flavonoids estimation, maximum levels found in IBA supplemented medium with 42.16 ± 2.31 (mg QE/g dw) followed by IAA hormone supplemented callus with levels of 29.00 ± 2.56 mg QE/g dw (Table 1). On compound quantification, *β*-sitosterol and caffeic acid were estimated in pet. ether and methanol extracts, respectively. Higher levels of *β*-sitosterol and caffeic acid were found in Kn hormone grown callus (0.198 and 4.42 mg/gdw of extract respectively).

**Table 1:** Yield %, total phenolics and flavonoids levels of callus extracts

**MS+GH**	**Type of extract**	**%Yield**	Total Phenolics (GAE/ g dw)*	**Total Flavonoids (QE/ g dw)** **+**	**Concentration (mg/gdw of extract)**	
					*β* **- sitosterol**	**Caffeic acid**
IAA	PE	1.44	*NA	NA	0.168	-
	Met	6.19	96.66 ± 7.47	29.00 ± 2.56	-	3.60
	Aq	5.53	65.33 ± 6.40	18.00 ± 3.51	-	-
IBA	PE	1.83	NA	NA	0.190	-
	Met	4.64	100.00 ± 5.78	42.16 ± 2.31	-	3.54
	Aq	6.52	90.00 ± 2.31	7.33 ± 0.83	-	-
Kn	PE	1.27	NA	NA	0.198	-
	Met	7.87	107.33 ± 1.76	19.83 ± 1.59	-	4.42
	Aq	4.21	60.00 ± 4.16	14.66 ± 2.33	-	-

PE-Pet. ether; Met-Methanol; Aq-Aqueous extracts; *NA-not quantified due to the poor quantity. MS+GH = Murashige and Skoog’s medium + growth hormone. *GAE/g dw = Gallic acid equivalent/g dry weight.+QE/g dw = Quercetic equivalent/g dry weight

Active principle was identified and confirmed as *β*-sitosterol [Rf; 0.06, I2 vapour (brown); color after spraying with 10% H2SO4 (purple) and 10% SbCl3 (blue)] and caffeic acid [Rf; 0.73 ammonia vapour (yellow), iodine vapour (brown); color after spraying with FC reagent (purple) and 5% methanolic AlCl3 (dull yellow)]. Compounds were confirmed on the basis of m.p. and IR spectral studies (Table 2).

**Table 2:** Chromatographic behavior and chemical characteristics of isolated compounds from *S. pauciflorum *cell cultures

**Isolated compounds**	**R** **F ** **(× 100)**		**Color after spray**		**m.p.(°C)**	**IR (ν ** **max** **) cm** **-1 ** **(KBr)**
	I	II	I	II		
*β*-Sitosterol	06	-	Blue	-	136-137	1730, 1640, 1240, 735, 725
Caffeic acid	-	76	-	Yellow	210-121	812, 849, 899, 972, 1118, 1172, 1212, 1448, 1640, 3440

I: Heptane-benzene-alcohol (100:100:1), sprayed with 10% SbCl3; II: Butanol: 27% aqueous acetic acid (1:1 v/v); sprayed with 10% methanolic AlCl3.

Pet. ether and methanol demonstrated appreciable antimicrobial activities in all tested extracts, whereas the pet. ether fraction of IBA callus found to be more effective against both the test bacteria and fungi (*S. aureus *IZ 14.00 ± 0.57 mm and *T. rubrum *16.33 ± 0.32 mm; Table 3), followed by its methanol extract (*S. aureus *IZ 13.00 ± 0.57 mm, *A. niger *and *P. chrysogenum *IZ 16.66 mm in both). IAA regenerated callus methanol extract also showed appreciable antimicrobial activity (*S. aureus *IZ 16.66 ± 0.88 mm, *C. albicans *IZ 15.33 ± 0.32 mm and *T. rubrum *17.66 ± 0.88 mm).

MS+GH = Murashige and Skoog’s medium + growth hormone.

In case of antioxidant activity, aqueous extracts were more active whereas IBA and Kn grown callus extracts demonstrated 0.06 mg/mL IC50 value (%inhibition 93.30 and 92.70 respectively at 0.8 mg/mL concentration; Table 4). Likewise, pet. ether fraction of IBA and Kn which had 0.07 mg/mL IC50 value (with % inhibition of 80.21 and 81.57 respectively at 0.08 mg/mL concentration). In FRAP method, the maximum antioxidant potential was demonstrated by methanolic extract of Kn grown callus 383.8 ± 6.67 at 1000 μg/mL concentration.

**Table 3:** Antimicrobial activity of *S. pauciflorum *stem callus at different growth hormone

**MS+GH Extract**		** Bacteria**						**Fungi**					
			***E. aerogenes***	***E. coli***	***P. aeruginosa***	***R. planticola***	***S. aureus***		***A. flavus***	***A. niger***	***C. albicans***	P.chrysogenum	***T. rubrum***
IAA IBA Kn *In-vivo *plant	PE Met Aq PE Met Aq PE Met Aq PE Met Aq	IZa	10.6 ± 0.3	10.3 ± 0.3	10.0 ± 0.0	10.00 ± 0.00	12.0 ± 0.0		-	10.3 ± 0.3	13.0 ± 0.5	13.3 ± 0.3	17.0 ± 0.5
		AIb	0.76	0.54	0.50	0.45	0.57		-	0.38	0.59	0.63	0.58
		IZ	13.3 ± 0.3	14.6 ± 1.2	11.0 ± 0.5	11.33 ± 0.33	16.6 ± 0.8		8.0 ± 0.0	10.0 ± 0.0	15.3 ± 0.3	13.3 ± 0.3	17.6 ± 0.8
		AI	0.95	0.77	0.55	0.51	0.79		0.29	0.37	0.69	0.63	0.60
		IZ	10.0 ± 0.5	10.0 ± 0.0	-	-	13.6 ± 0.3		-	-	-	-	-
		AI	0.71	0.52	-	-	0.65		-	-	-	-	-
		IZ	13.6 ± 0.3	13.6 ± 0.3	12.3 ± 0.3	11.33 ± 0.67	14.0 ± 0.5		13.3 ± 0.8	12.3 ± 0.6	16.0 ± 0.0	13.6 ± 0.6	16.3 ± 0.3
		AI	0.97	0.71	0.61	0.51	0.66		0.49	0.45	0.72	0.63	0.56
		IZ	11.6 ± 0.8	11.6 ± 0.8	12.0 ± 0.0	12.00 ± 0.81	13.0 ± 0.5		10.6 ± 0.6	16.6 ± 0.8	13.3 ± 0.6	16.6 ± 0.3	8.0 ± 0.0
		AI	0.83	0.61	0.60	0.54	0.61		0.50	0.61	0.60	0.79	0.29
		IZ	10.0 ± 0.0	10.0 ± 0.0	11.6 ± 0.3	10.00 ± 0.00	10.3 ± 0.3		-	-	-	-	-
		AI	0.71	0.52	0.58	0.45	0.49		-	-	-	-	-
		IZ	10.3 ± 0.3	10.3 ± 0.3	14.0 ± 0.5	11.66 ± 0.66	10.0 ± 0.0		11.0 ± 0.5	9.3 ± 0.8	12.0 ± 0.5	13.3 ± 0.6	12.6 ± 0.6
		AI	0.73	0.54	0.70	0.53	0.47		0.40	0.34	0.54	0.63	0.43
		IZ	10.3 ± 0.3	-	13.6 ± 0.8	11.66 ± 0.3	13.6 ± 0.8		13.0 ± 0.5	10.0 ± 0.0	13.3 ± 0.3	14.6 ± 0.6	12.3 ± 0.3
		AI	0.73	-	0.68	0.53	0.65		0.48	0.37	0.60	0.69	0.43
		AI	13.3 ± 0.7	8.0 ± 0.0	15.3 ± 0.3	9.0 ± 0.5	9.0 ± 0.5		-	-	-	-	-
		IZ	0.96	0.42	0.75	0.40			-	-	-	-	-
		AI	13.0 ± 0.5	11.6 ± 0.6	14.6±0.3	11.3 ± 0.3	8.0 ± 0.0		12.6 ± 0.6	11.0 ± 0.3	12.6 ±0.6	11.6 ± 0.3	10.0 ± 0.0
		IZ	0.92	0.61	0.34	0.51	0.38		0.46	0.40	0.57	0.55	0.34
		AI	13.0 ± 0.5	10.6 ± 0.3	13.3 ± 0.3	8.6 ± 0.6	12.0 ± 0.5		10.0 ± 1.0	10.0 ± 0.0	11.3 ± 0.6	11.6 ± 0.3	10.0 ± 0.0
		IZ	0.92	0.56	0.66	0.39	0.57		0.37	0.37	0.51	0.56	0.34
		AI	10.3 ± 0.3	10.3 ± 0.3	15.6 ± 0.6	10.3 ± 0.3	10.3 ± 0.3		10.0 ± 0.0	12.6 ± 0.7	11.6±0.3	13.3 ± 0.3	9.3 ± 0.3
		IZ	0.73	0.54	0.78	0.46	0.49		0.37	0.46	0.53	0.63	0.34

Antimicrobial activity (in terms of inhibition zone in mm including the diameter of well; 6 mm); Mean ± SE (Standard error); Standard = Streptomycin (10 mcg/mL) for bacteria, Ketonocozole, (10 mcg/disc) for fungi; PE = pet. ether, Met = methanol, Aq = aqueous.

**Table 4:** Total phenolics and antioxidant assay by DPPH and FRAP method

**MS+GH**	**Extracts**	**IC50**	*% Inhibition (conc. in mg/mL)					**+AAE/ mg dw (conc. in μg/mL)**				
			**0.1**	**0.2**	**0.4**	**0.6**	**0.8**	**62.5**	**125**	**250**	**500**	1000
IAA	PE	2.00	45.33	50.37	57.85	58.49	66.64	216.2 ± 3.37	30.4 ± 0.00	236.2 ± 8.25	280.5 ± 10.0	346.6 ± 16.6
	Met	0.10	49.34	65.03	69.02	72.75	75.65	270.1 ± 0.00	273.2 ± 3.19	283.6 ± 3.19	293.1 ± 3.19	356.8 ± 3.37
	Aq	0.09	53.53	57.62	62.38	65.29	68.00	310.6 ± 0.00	320.7 ± 0.00	323.2 ± 6.60	340.1 ± 8.13	356.7 ± 6.69
IBA	PE	0.07	68.46	69.63	72.34	78.60	80.21	290.5 ± 0.00	303.9 ± 3.37	320.4 ± 0.00	336.5 ± 5.89	373.3 ± 3.37
	Met	1.55	28.90	32.68	68.10	72.80	80.32	266.5 ± 3.37	273.5 ± 3.19	290.5 ± 0.00	310.8 ± 0.00	336.3 ± 8.45
	Aq	0.06	82.95	87.24	92.40	92.74	93.30	263.9 ± 3.80	290.2 ± 0.00	303.1 ± 3.34	313.6 ± 3.34	366.4 ± 6.69
Kn	PE	0.07	74.29	76.59	76.84	81.16	81.57	204.1 ± 7.21	221.4 ± 4.77	290.9 ± 0.00	313.5 ± 3.34	323.1 ± 12.0
	Met	0.08	65.16	69.02	72.57	77.12	79.73	263.4 ± 8.80	290.1 ± 0.00	303.1 ± 3.34	313.1 ± 3.34	383.8 ± 6.67
	Aq	0.06	82.59	83.54	90.90	92.69	92.70	280.8 ± 0.00	286.6 ± 3.37	300.8 ± 0.00	300.3 ± 0.00	343.2 ± 3.34
*In-vivo *plant	PE	-	68.61	70.61	70.61	74.40	73.75	28.3 ± 4.41	21.0 ± 6.69	25.0 ± 0.00	31.6 ± 3.78	46.6 ± 3.33
	Met	0.15	47.32	51.80	72.60	72.93	80.04	110.0 ± 3.33	211.6 ± 7.20	220.0 ± 1.95	275.0 ± 0.00	351.6 ± 9.36
	Aq	0.075	68.48	76.92	92.51	93.40	93.78	32.5 ± 5.66	32.6 ± 4.56	43.9 ± 3.33	51.9 ± 3.48	51.1 ± 5.69

PE = pet. ether, Met = methanol, Aq = aqueous. * %Inhibition = 1 - (Absorbance of the sample/Absorbance of the control) × 100.+AAE/ mg dw = ascorbic acid equivalent/mg dry weight. MS+GH = Murashige and Skoog’s medium + growth hormone.

In a plant cell or tissue culture study, growth regulator study plays a crucial role in secondary metabolites accumulation (15). The concentration of auxin and cytokinin individually or in combination significantly alters both the growth and secondary metabolite accumulation in cultures cells (16). In auxins, naphthalene acetic acid (NAA) or IAA enhanced the production of nicotine in suspension on *Nicotiana tabacum *and shikonin in suspension cultures of *Lithospermum erthrorhizon *(17-18). Cytokinins like Kinetin has been found to stimulate the production of anthocyanin in *Haplopappus gracilis *cell culture (19).

In the present experiment, production of *β*-sitosterol and caffeic acid was investigated with influence of different growth hormones. Higher levels of phenolics and flavonoids were found in Kn grown callus. In a similar way, caffeic acid was estimated maximum in the same callus extract. IBA grown callus also effectively produced secondary metabolites. In antimicrobial activities, pet. ether extract of IBA grown cell callus demonstrates appreciable activity as it shows more levels of total flavonoids and higher amounts of phenolics. In case of antioxidant activity, IBA and Kn grown callus demonstrates maximum inhibition with coinciding the levels of phenolics and flavonoids present in the callus. Similarly, FRAP method also showed similar behaviour where Kn grown callus demonstrated higher antioxidant potentials.

From the present study, it is evidenced that plant callus is effective in enhancing the production of secondary metabolites at a low concentration of growth hormones. Cytokinins are known to enhance the production of secondary metabolites and play an important role in cytodifferentiation (20) and subcellular differentiation, *e.g*., anthocyanin production in *Camptotheca acuminata *(21). Modulation of secondary metabolites production by plant growth regulators is very old (22), but the marked effect of production of terpenoids and phenolics with auxins and cytokinin in callus and cell cultures of *Commiphora wightii *and isoflavonoids in cell cultures of *Pueraria tuberosa *(23) was reported earlier.

Therefore, the protocol developed under this study was superior for the production of secondary metabolites compared with the *in-vivo *plant. IBA regenerated callus proved to be better for the accumulation of caffeic acid and *β*-sitosterol. Secondary metabolites demonstrated variability in their levels amount that could be helpful in a wide array of health promoting those benefits in antioxidants, anti-inflammatory and anticancerous agents.
